# ISGF3 and STAT2/IRF9 Control Basal and IFN-Induced Transcription through Genome-Wide Binding of Phosphorylated and Unphosphorylated Complexes to Common ISRE-Containing ISGs

**DOI:** 10.3390/ijms242417635

**Published:** 2023-12-18

**Authors:** Hanna Nowicka, Agata Sekrecka, Katarzyna Blaszczyk, Katarzyna Kluzek, Chan-Yu Chang, Joanna Wesoly, Chien-Kuo Lee, Hans A. R. Bluyssen

**Affiliations:** 1 Human Molecular Genetics Research Unit, Faculty of Biology, Institute of Molecular Biology and Biotechnology, Adam Mickiewicz University, 61-614 Poznan, Poland; 2 Graduate Institute of Immunology, National Taiwan University College of Medicine, Taipei 100, Taiwan; 3 Laboratory of High Throughput Technologies, Faculty of Biology, Adam Mickiewicz University, 61-614 Poznan, Poland

**Keywords:** interferon type-I, JAK/STAT signaling, ISGF3, U-ISGF3, STAT2/IRF9, U-STAT2/IRF9, integrative omics approach, IFN-dependent and -independent transcription

## Abstract

In addition to the canonical ISGF3 and non-canonical STAT2/IRF9 complexes, evidence is emerging of the role of their unphosphorylated counterparts in IFN-dependent and -independent ISG transcription. To better understand the relation between ISGF3 and U-ISGF3 and STAT2/IRF9 and U-STAT2/IRF9 in IFN-I-stimulated transcriptional responses, we performed RNA-Seq and ChIP-Seq, in combination with phosphorylation inhibition and antiviral experiments. First, we identified a group of ISRE-containing ISGs that were commonly regulated in IFNα-treated WT and STAT1-KO cells. Thus, in 2fTGH and Huh7.5 WT cells, early and long-term IFNα-inducible transcription and antiviral activity relied on the DNA recruitment of the ISGF3 components STAT1, STAT2 and IRF9 in a phosphorylation- and time-dependent manner. Likewise, in ST2-U3C and Huh-STAT1KO cells lacking STAT1, delayed IFN responses correlated with DNA binding of phosphorylated STAT2/IRF9 but not U-STAT2/IRF9. In addition, comparative experiments in U3C (STAT1-KO) cells overexpressing all the ISGF3 components (ST1-ST2-IRF9-U3C) revealed U-ISGF3 (and possibly U-STAT2/IRF9) chromatin interactions to correlate with phosphorylation-independent ISG transcription and antiviral activity. Together, our data point to the dominant role of the canonical ISGF3 and non-canonical STAT2/IRF9, without a shift to U-ISGF3 or U-STAT2/IRF9, in the regulation of early and prolonged ISG expression and viral protection. At the same time, they suggest the threshold-dependent role of U-ISFG3, and potentially U-STAT2/IRF9, in the regulation of constitutive and possibly long-term IFNα-dependent responses.

## 1. Introduction

As the main mediators of cellular homeostatic responses to viral infection, Type I Interferons (IFN-I) are produced by many different cell types. IFN-I predominantly consist of the IFNα and IFNβ subtypes, which target responsive cells by interacting with the heterodimeric transmembrane IFN-I receptor (IFNAR). This activates members of the Janus kinase (Jak) and signal transducer and activator of transcription (STAT) family and the JAK/STAT signaling cascade. In the canonical IFN-I-mediated signaling pathway, Jak1 and Tyk2 phosphorylate STAT1 on Tyr701 and STAT2 on Tyr690, which after heterodimerization interact with IFN Regulatory Factor 9 (IRF9) and form IFN Stimulated Gene Factor 3 (ISGF3). Subsequently, this complex translocates to the nucleus and activates the transcription of numerous IFN Stimulated Response Element (ISRE)-containing antiviral IFN-stimulated genes (ISGs) [1,2,3]. 

Recently, evidence has emerged of the existence of a non-canonical IFN-I signaling pathway, in which the ISGF3-like complex STAT2/IRF9 was shown to activate transcription of ISRE-containing genes in response to IFNα in the absence of STAT1 [4]. Under these conditions, the IFNα-induced expression of typical antiviral ISGs correlated with the kinetics of STAT2 phosphorylation and the presence of a STAT2/IRF9 complex. More importantly, the STAT2/IRF9 complex triggered the expression of a similar subset of ISGs as ISGF3, although with a more prolonged expression profile [1,5,6]. As a consequence, STAT2/IRF9 was able to induce an antiviral response upon encephalomyocarditis virus (EMCV) and vesicular stomatitis Indiana virus (VSV). Different in vitro and in vivo studies have subsequently pointed to the existence of a STAT1-independent IFN-I signaling pathway, where STAT2/IRF9 can potentially substitute for the role of ISGF3 [7,8,9,10,11].

In accordance with the general paradigm of IFN-I signaling, a robust and transient phosphorylation pattern of STAT1 and STAT2 is followed by a similar ISG expression profile that decreases over time. Conversely, recent studies revealed more complexity of this response, with more prolonged ISG expression patterns that were shown to correlate with a drop in STAT phosphorylation and rely on sustained expression of the components of ISGF3 as part of a positive feedback loop [2,12,13,14]. In this context, an ISGF3-like complex has been reported, composed of unphosphorylated STAT1 and STAT2 with IRF9 (named U-ISGF3), which may switch with ISGF3 to drive prolonged expression of a subset of U-ISGs in response to IFN-I [12,13]. U-ISGs are exclusively regulated by ISGF3 at early time points, and further induced by U-ISGF3 at late time points, to maintain the long-term IFN-I response. Likewise, long-term IFN responses in the absence of STAT1 have been shown to depend on expression of STAT2 and IRF9, with a possible regulatory role of U-STAT2/IRF9 in prolonged ISG transcription [2]. Moreover, evidence exists that U-ISGF3 [15] and U-STAT2/IRF9 [16] can be formed independently of IFN-I treatment and regulate basal ISG expression. Accordingly, Platanitis [16] proposed the presence of a molecular switch from STAT2/IRF9 to ISGF3 that underlies IFN-induced transcription in mouse cells, but not in humans.

In this study, we addressed the following objectives: 1. To understand in more detail the role of ISGF3 vs. U-ISGF3 and STAT2/IRF9 vs. U-STAT2/IRF9 in long-term IFN-I-stimulated transcriptional responses. 2. To assess the role of U-ISGF3 and U-STAT2/IRF9 in phosphorylation-independent ISG transcription and antiviral activity under basal and IFN-induced conditions. For this, we performed RNA-Seq and ChIP-Seq, in combination with phosphorylation inhibition and antiviral experiments in WT, STAT1-KO and STAT1, STAT2 and IRF9-overexpressing cells. Collectively, our data point to the dominant role of the canonical ISGF3 and non-canonical STAT2/IRF9 in the regulation of early and prolonged ISG expression and viral protection. Our data disagree with a model in which U-ISGF3 or U-STAT2/IRF9 may switch with ISGF3 or STAT2/IRF9 to drive prolonged expression of U-ISGs in response to IFN-I. However, they are in favor of an additional, threshold-dependent role of U-ISFG3, and possibly U-STAT2/IRF9, in the regulation of constitutive and possibly long-term IFN-dependent ISG expression.

## 2. Results

### 2.1. Characterization of Time-Dependent IFNα Responses in WT and STAT1-KO Cells 

**Figure 1:**
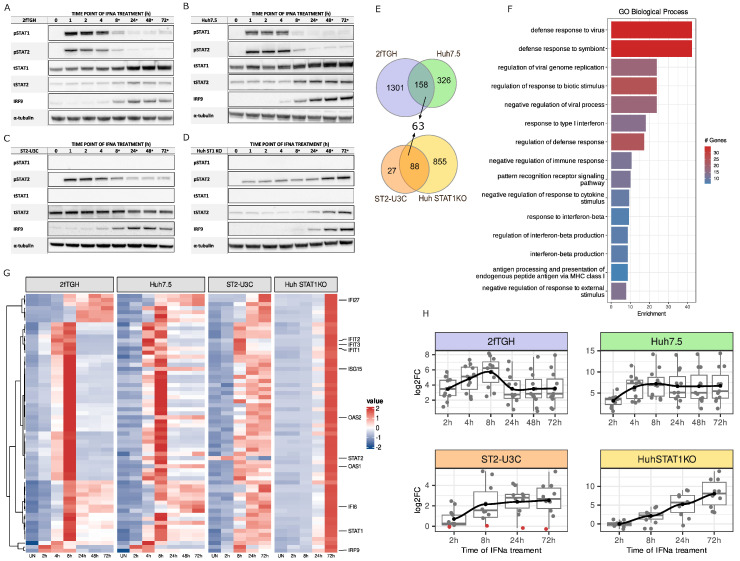
ISGF3 and STAT2/IRF9 regulate transcription of a common group of ISRE-containing genes in a phosphorylation- and time-dependent manner. (**A**–**D**) Protein synthesis and phosphorylation patterns in IFNα-treated 2fTGH, Huh7.5, ST2-U3C and Huh STAT1KO cells evaluated via immunoblotting; p—phosphorylated, t—total, *—late phase of IFNα response. (**E**) The 63 genes commonly upregulated in WT (2fTGH and Huh7.5) vs. KO (ST2-U3C and Huh STAT1KO) cell lines are presented using a Venn diagram, based on the RNA-Seq results of 2fTGH (blue), Huh7.5 (green), ST2-U3C (orange) and Huh STAT1KO (yellow), log_2_FC > 1 or 0.5 (see Material and Methods); padj < 0.05. (**F**) Gene Ontology terms enrichment analysis of 63 commonly upregulated genes in 2fTGH, Huh7.5, ST2-U3C and Huh STAT1KO (significant enrichment considered with FDR < 0.05). (**G**) Heatmaps generated from the expression values of commonly upregulated genes in 2fTGH, Huh7.5, ST2-U3C and Huh STAT1KO. Counts were normalized using the Z-score. Each row represents one of 63 genes, preselected ISGs are highlighted. (**H**) Boxplots representing the expression profiles of the pre-selected 11 commonly upregulated genes (see text) in 2fTGH (blue), Huh7.5 (green), ST2-U3C (orange) and Huh STAT1KO (yellow), log_2_FC > 1 or 0.5 and padj < 0.05. The black lines represent mean log_2_FC value for each time point. The red dots indicate the STAT2 gene.

To study the role of the phosphorylation of ISGF3 and STAT2/IRF9 in long-term IFN-I signaling, we first characterized the IFNα-induced expression and phosphorylation of the ISGF3 components at the protein level in 2fTGH and Huh7.5 WT cells (Figure 1A–D). Therefore, cells were treated with IFNα for 0, 1, 2, 4, 8, 24, 48 and 72 h. In 2fTGH cells, phosphorylation of STAT1 and STAT2 was absent in untreated controls but highly induced upon IFNα treatment with an early, transient increase between 1 and 4 h, after which it rapidly diminished to still detectable levels at 72 h (Figure 1A). A similar early and transient pattern of IFNα-dependent STAT1 and STAT2 phosphorylation was observed in Huh7.5 cells (Figure 1B), with a clear drop between 4 and 8 h after treatment. Likewise, in these cells, the phosphorylation of STAT1 and STAT2 was still clearly visible after 72 h. In contrast to their phosphorylated counterparts, expression of unphosphorylated STAT1 (U-STAT1) and U-STAT2 was already detectable in untreated 2fTGH and Huh7.5 cells, and further induced between 4 and 72 h after IFNα. The same pattern was apparent for IRF9, although it was nearly undetectable in resting 2fTGH and below the Western blot detection level in untreated Huh7.5 cells. As part of the late and long-term response, the IFNα-induced expression of U-STAT1, U-STAT2 and IRF9 in both cell types followed the drop in phosphorylation of STAT1 and STAT2 between 8 and 72 h (Figure 1A,B). This correlated with the positive feedback regulation of the ISGF3 components observed in response to IFNα. Also, it may lead to the formation of U-ISGF3 and the potential involvement of this complex in mediating the prolonged IFN Type-I signaling [12].

Likewise, we treated the STAT1-deficient cell lines ST2-U3C [5] and Huh STAT1KO [9,14] with IFNα to examine the role of STAT2/IRF9 in the absence of STAT1 (Figure 1C,D). In the case of ST2-U3C (Figure 1C), a subtle change in the STAT2 phosphorylation profile could be observed as compared to 2fTGH cells (Figure 1A). The profile remained transient, with an early increase between 1 and 2 h, but in contrast to wild-type cells, the level of pSTAT2 only decreased slowly and remained still high until 72 h. This also corresponded to a rise in IRF9 expression in ST2-U3C at later time points, being nearly undetectable in resting cells, and it pointed to a delayed formation of STAT2/IRF9 in these cells as compared to ISGF3 in 2fTGH. As STAT2 is overexpressed in ST2-U3C cells, STAT2 expression was not affected by IFN treatment. Different from ST2-U3C, Huh STAT1KO cells (Figure 1D) displayed a rather shifted STAT2 phosphorylation pattern, being delayed and prolonged as compared to Huh7.5 (Figure 1B). Accordingly, in Huh STAT1KO, the pSTAT2 levels reached a maximum after 72 h of INFα treatment. Like in ST2-U3C, in Huh STAT1KO cells, IFN-dependent accumulation of IRF9 (not detectable in untreated cells) and also U-STAT2, was observed; however, it predominantly occurred at later time points. How this depends on the phosphorylation of STAT2 and if a contribution of U-STAT2/IRF9 under these conditions exists have not been studied. 

### 2.2. Genome-Wide Characterization of IFNα-Induced Transcription in WT vs. STAT1KO Cells 

Next, we performed RNA-Seq on RNA from 2fTGH, Huh7.5, ST2-U3C and HuhSTAT1KO treated with IFNα for different time points (see Material and Methods). Differential gene expression (DEG) analysis was performed and genes upregulated during at least one of the time points were selected based on the cut-off of log_2_FC > 1 and padj < 0.05. As such, IFNα-induced expression of 1459 genes in 2fTGH and 484 in Huh7.5 cells, of which 158 genes were in common (Figure 1E). In ST2-U3C and Huh STAT1KO, IFNα induced expression of 115 and 943 genes, respectively, of which 88 genes were in common (Figure 1E). When compared to the 158 commonly IFNα-induced genes from 2fTGH vs. Huh7.5, a group of 63 IFNα-inducible genes showed overlap between WT and STAT1 deficient cell lines (Figure 1E). 

GO term enrichment analysis of the 63 common genes revealed significant enrichment in biological terms connected to Type-I IFN signaling as well as broadly defined antiviral responses, and it recognized these genes among the core subset of antiviral ISGs (Figure 1F). The expression profiles of the 63 common genes in IFNα-treated 2fTGH, Huh7.5, ST2-U3C and Huh STAT1KO cells are presented in the form of a heatmap in Figure 1G. Not surprisingly, a transient expression pattern was observed in both WT cell lines (2fTGH vs. Huh7.5) upon IFNα treatment, with a maximum at 8 h, followed by a significant drop to still detectable levels beyond 24 h. On the other hand, STAT1 deficiency changed their expression profile dramatically, resulting in lower and prolonged expression in the case of ST2-U3C, and delayed and prolonged expression in the case of Huh STAT1KO (Figure 1G). More importantly, the gene expression patterns of the 63 common IFNα-induced genes in these four different cell lines reflect the phosphorylation profiles of STAT1 and/or STAT2, as described above (Figure 1A–D). 

Closer examination of this group of 63 genes allowed for the identification of many known ISRE-containing antiviral ISGs (Figure 1G), including *IFIT1*, *IFIT2*, *IFIT3*, *ISG15*, *IFI6*, *IFI27*, *OAS1*, and *OAS2*, as well as the components of ISGF3 (*STAT1*, *STAT2* and *IRF9*). After plotting the mean log_2_FC for each time point, in 2fTGH cells, the pre-selection of these 11 genes exhibited a similar IFN-induced expression profile, with a maximum expression level after 8 h of treatment, followed by a significant drop to still detectable levels at 72 h (Figure 1H). In contrast, in Huh7.5 cells, the mean expression profile of these genes was more prolonged, reaching maximum expression between 8 and 72h (Figure 1H). Also, the potency of the transcriptional responses in Huh7.5 was higher in comparison to 2fTGH cells. In ST2-U3C, the mean expression profile of these pre-selected ISGs was much lower and their expression profile more prolonged as compared to 2fTGH cells (Figure 1H). As STAT2 is overexpressed in ST2-U3C cells, STAT2 gene expression differences between different time points cannot be measured (STAT2 expression profile marked as red dots). Likewise, in Huh STAT1KO, the mean expression profile of these ISGs was prolonged, but in addition, even more delayed, shifting the expression profiles of all the selected ISGs toward later time points while reaching comparable levels as in Huh7.5 cells (Figure 1H). This also further proves that in Huh STAT1KO cells, IFNα-mediated ISG expression clearly depends on both STAT2 and IRF9. With the IRF9 amounts in untreated Huh STAT1KO cells below the WB detection level, the early presence of STAT2 is not sufficient to trigger significant gene expression and strongly delays the start of transcription until higher amounts of IRF9 are reached. To validate the quality of our RNA-Seq dataset in general and the expression profiles observed for the pre-selected ISRE-containing antiviral ISGs, the expression of OAS2, IFIT1, IFI27 and IFI6 was additionally confirmed via qPCR and compared in the four different cell lines (Figure S1). 

Together, these results provide evidence to suggest that ISGF3 and STAT2/IRF9 regulate transcription of a common group of ISRE-containing genes in a phosphorylation- and time-dependent manner. They also confirm the previous observation that STAT2/IRF9 can take over the role of ISGF3 and generate an antiviral response in the absence of STAT1 [5].

### 2.3. Genome-Wide Binding of Phosphorylated and Unphosphorylated ISGF3 Components to ISRE Sites of IFNα Upregulated Genes in WT vs. STAT1KO Cells 

**Figure 2:**
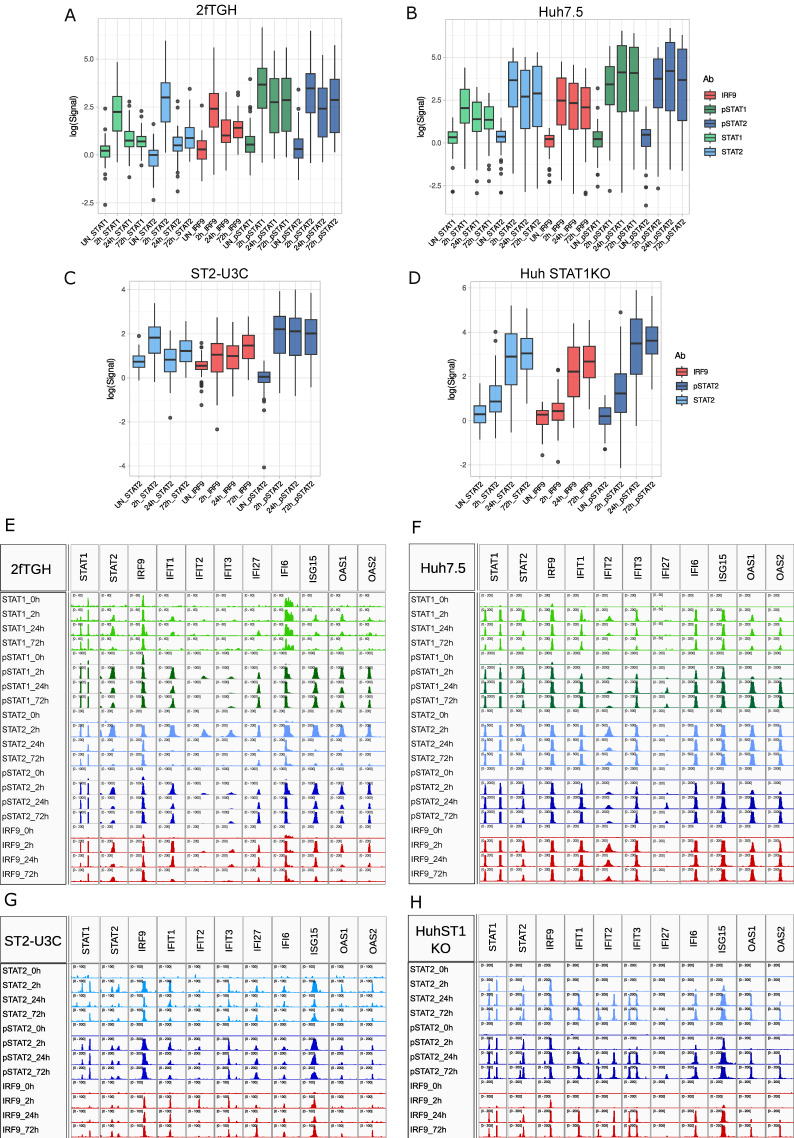
Genome-wide promoter interactions of phosphorylated and unphosphorylated ISGF3 and STAT2/IRF9 components reflect the ISG transcription profiles. (**A**–**D**) ChIP-Seq peak distribution for STAT1 (light green), pSTAT1 (dark green), STAT2 (light blue), pSTAT2 (dark blue) and IRF9 (red) in IFNα-treated 2fTGH (**A**), Huh7.5 (**B**), ST2-U3C (**C**) and Huh STAT1KO (**D**) presented as boxplots showing the average peak signal generated using Macs2 (corresponding values—left y axis). (**E**–**H**) Representative ChIP peaks graphically presented using IGV software (color coding corresponds to **A**–**D**). Peak scale was set as follows: (**E**) STAT1 0–60, pSTAT1 0–1000, STAT2 0–200, pSTAT2 0–1000, IRF9 0–200, (**F**) STAT1 0–200, pSTAT1 0–2000, STAT2 0–500, pSTAT2 0–2000, IRF9 0–200, except for IFI27 were for STAT1 scale is 0–50, and 0–200 for other antibodies, (**G**) STAT2 0–100, pSTAT2 0–200, IRF9 0–100 and (**H**) STAT2 0–200, pSTAT2 0–200, IRF9 0–200; p—phosphorylated.

We subsequently characterized the genome-wide binding of phosphorylated and unphosphorylated ISGF3 components to the regulatory regions of the 63 common IFNα-induced genes in WT and STAT1KO cells (Figure 2). ChIP-Seq was performed on chromatin isolated from cells treated with IFNα for 0, 2, 24 and 72 h, using antibodies against STAT1, STAT2, pSTAT1, pSTAT2 and IRF9 (2fTGH and Huh7.5) and STAT2, pSTAT2 and IRF9 (ST2-U3C and Huh STAT1KO). After statistical analysis (Table S1), under these conditions, the peak number distribution in 2fTGH followed a transient pattern for all the antibodies, with a maximum at 2 h of IFNα stimulation and no basal binding (Figure 2A, Table S1). Moreover, STAT1, STAT2, pSTAT1, pSTAT2 and IRF9 binding peaks could still be detected after 72 h. The peak number distribution in Huh7.5 cells exhibited a higher potency and a more prolonged pattern for all the antibodies, with high binding scores at 2, 24 and 72 h of IFNα stimulation (Figure 2B, Table S1). Binding was absent in untreated Huh7.5 cells, whereas no shift could be detected from the binding of phosphorylated STAT1 and STAT2 to U-STAT1 and U-STAT2 at later time points. Collectively, the IFNα- and time-dependent distribution of STAT1, STAT2, pSTAT1, pSTAT2, and IRF9 binding in 2fTGH and Huh7.5 correlated with the expression profile observed for the 63 common IFNα-induced genes in the individual cell lines (see Figure 1G,H). The peak number distribution in ST2-U3C and Huh STAT1KO cells followed a prolonged pattern for all the antibodies, without binding in untreated cells. The maximal peak number in ST2-U3C was observed at 2 h (Figure 2C; Table S1), while in Huh STAT1KO, it was shifted toward 24 h (Figure 2D; Table S1). Moreover, in both cell lines, STAT2, pSTAT2 and IRF9 binding peaks could still be detected after 72 h, without a detectable shift from phosphorylated STAT2 to U-STAT2 after long-term treatment. Additionally, the peak scores in ST2-U3C were generally much lower than in Huh STAT1KO, which correlated with the gene expression pattern observed in these two cell lines (see Figure 1G,H). 

Successively, using HOMER software, the ISRE and GAS consensus motifs (Figure S2) were mapped to the IFNα-induced STAT1, STAT2, pSTAT1, pSTAT2 and IRF9 binding regions of the 63 common IFNα-induced genes. Not surprisingly, all of these genes contained an ISRE binding site (not shown). Closer inspection of the 11 pre-selected genes (Figure 1G,H) confirmed a correlation between the gene expression and recruitment of STAT1, STAT2, pSTAT1, pSTAT2 and IRF9 in response to IFNα in 2fTGH (more transient, Figure 2E) and Huh7.5 (more prolonged, Figure 2F), and STAT2, pSTAT2 and IRF9 in ST2-U3C (more prolonged, Figure 2G) and HuhSTAT1KO (delayed, Figure 2H). Unfortunately, the peak scores for all the antibodies for the IFIT2 gene were much lower in 2fTGH and ST2-U3C as compared to Huh7.5 and Huh ST1KO cells, whereas the opposite was true for IFI27. More surprisingly, for the *STAT1*, *IRF9* and *IFI6* genes’ binding of STAT1 and pSTAT1 was observed in untreated 2fTGH cells. As a result, *STAT1*, *IRF9* and *IFI6* genes showed a more potent and prolonged binding pattern for all the antibodies upon IFNα treatment of 2fTGH cells, similar to Hu7.5 cells (compare Figure 2E,F). Since this basal binding was not detectable in Huh7.5 cells, and their gene expression profile is transient in 2fTGH cells, we believe this may be the result of an unspecific effect of the antibodies. Nevertheless, for the majority of the pre-selected genes, the binding profiles of the different antibodies resembled the peak distribution in the different cell lines, as shown in Figure 2A–D. Also, no basal binding of the U-ISGF3 and U-STAT2/IRF9 components could be observed and no shift could be detected from the binding of phosphorylated STATs to U-STATs after long-term treatment. 

### 2.4. U-ISG Expression Correlates with pSTAT1 and pSTAT2 Expression and Long-Term Binding in Wild Type and STAT1KO Cells 

**Figure 3:**
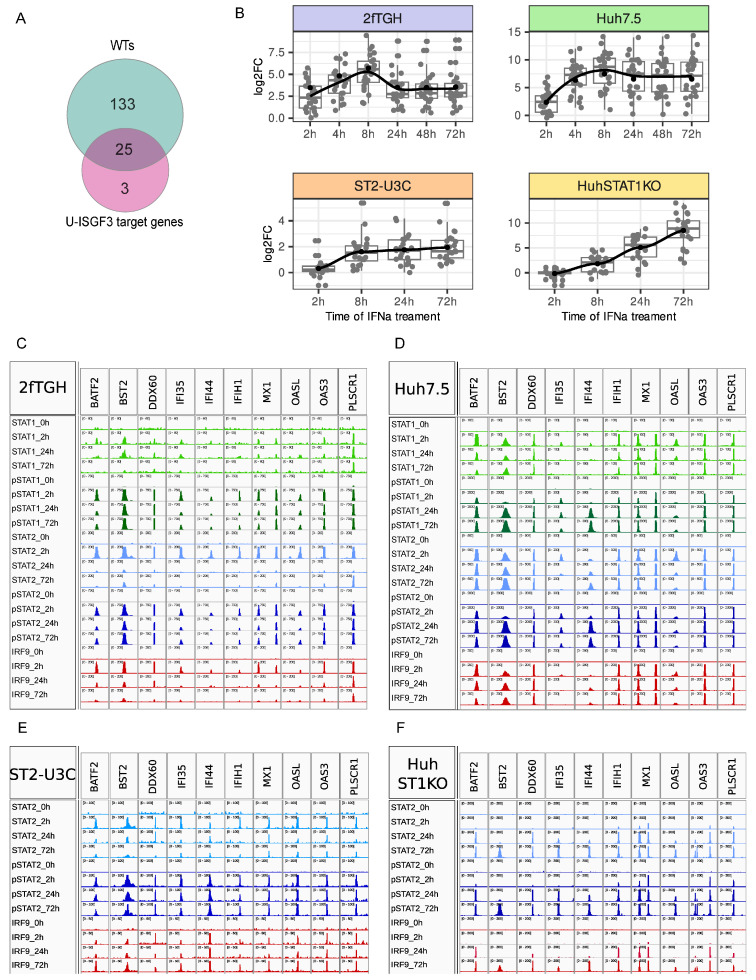
Long-term U-ISG expression profiles follow the chromatin binding patterns of pSTAT1 and pSTAT2. (**A**) Venn diagram showing the U-ISGF3 target genes described by Cheon et al. [12] (pink) upregulated in 2fTGH and Huh7.5 (WTs, turquoise). (**B**) RNA-Seq result-based boxplots representing the U-ISG expression profiles in 2fTHG (blue), Huh7.5 (green), ST2-U3C (orange) and Huh STAT1KO (yellow), log_2_FC > 1 or 0.5; padj < 0.05. (**C**–**F**) Binding of STAT1 (light green), STAT2 (light blue), pSTAT1 (dark green), pSTAT2 (dark blue) and IRF9 (red) to U-ISG promoters in 2fTGH (**C**), Huh7.5 (**D**), ST2-U3C (**E**) and Huh STAT1KO (**F**) demonstrated as ChIP peaks using IGV software. Peak scale was set as follows: (**C**) STAT1 0–60, pSTAT1 0–750, STAT2 0–200, pSTAT2 0–750, IRF9 0–200, (**D**) STAT1 0–100, pSTAT1 0–2000, STAT2 0–500, pSTAT2 0–2000, IRF9 0–200, except for IFI44 and IFI35 were the scale is 0–300 for pSTAT1 and pSTAT2 and 0–200 for other antibodies, (**E**) STAT2 0–100, pSTAT2 0–100, IRF9 0–50, (**F**) STAT2 0–200, pSTAT2 0–200, IRF9 0–200; p—phosphorylated.

According to Cheon et al. and Sung et al. [12,13], a subset of 29 ISRE-containing U-ISGs are exclusively regulated by ISGF3 at early time points, and further induced by U-ISGF3 at late time points, to maintain the long-term IFN-I response. When compared to the 158 common IFNα-induced genes from 2fTGH vs. Huh7.5, 25 of these U-ISGs were shown to be expressed in both cell lines (Figure 3A). Among these 25 genes, the presence of the 7 above characterized ISGs (compare Figure 1E–H) could be recognized, including *IFIT1*, *IFIT3*, *ISG15*, *IFI27*, *OAS1*, *OAS2*, and *STAT1* (Figure 1G,H). The remaining 18 genes included *DDX58*, *DDX60*, *BST2*, *IFI44*, *IFI44L*, *XAF1*, *IFITM1*, *RTP4*, *OAS3*, *IFIH1*, *HERC5*, *HERC6*, *MX1*, *PLSCR1*, *OASL*, *BATF2*, *TMEM140* and *IFI35*. 

As shown in Figure 3B, these 25 U-ISGs exhibited a similar IFN-induced expression profile in 2fTGH vs. Huh7.5, and in ST2-U3C vs. Huh-STAT1KO cells, which was comparable to the 63 common IFNα-induced genes (see Figure 1H). Indeed, a transient expression pattern was observed in both WT cell lines (2fTGH vs. Huh7.5) upon IFNα treatment, with a maximum at 8 h, followed by a significant drop to still detectable levels at 72 h. On the other hand, STAT1 deficiency changed their expression profile, resulting in lower and prolonged expression in the case of ST2-U3C, and delayed and prolonged expression in Huh STAT1KO cells (Figure 3B). Importantly, the gene expression patterns of the 25 U-ISGs in these four different cell types reflect the phosphorylation profiles and/or production of ISGF3 and STAT2/IRF9 components, as described above (Figure 1). 

Close comparison of the 7 previously characterized (Figure 1G,H) and the pre-selection of the remaining 18 U-ISGs (Figure 3C–F) confirmed a similar correlation between the gene expression and recruitment of STAT1, STAT2, pSTAT1, pSTAT2 and IRF9 in response to IFNα in 2fTGH (more transient, Figure 3C) and Huh7.5 (more prolonged, Figure 3D) cells, and STAT2, pSTAT2 and IRF9 in ST2-U3C (more prolonged, Figure 3E) vs. Huh STAT1KO (delayed, Figure 3F) cells. As an exception, the peak scores for the pSTAT1 and pSTAT2 antibodies for the IFI35 and IFI44 genes were much lower in Huh7.5 as compared to 2fTGH cells. However, in general for all 25 U-ISGs, the binding profiles of the different antibodies resembled the peak distribution, as shown in Figure 2, with STAT1, STAT2, pSTAT1, pSTAT2 and IRF9 binding peaks still detectable after 72 h. Also, no basal binding of the U-ISGF3 or U-STAT2/IRF9 components could be observed and no shift could be detected from the binding of phosphorylated STATs to U-STATs after long-term treatment. 

### 2.5. The Role of Phosphorylation of ISGF3 and STAT2/IRF9 in the Regulation of Prolonged ISG Expression in Wild Type and STAT1KO Cells 

To further address the role of the phosphorylation of STAT1 and STAT2, especially in the long-term IFN-I activated transcriptional responses of WT and STAT1KO cells (see Figure 1A,B), experiments with JAK Inhibitor I (JII) were performed. JII potently inhibits the activity of all four Janus kinases, and consequently, blocks the IFNα-dependent phosphorylation of STAT1 and STAT2. 

**Figure 4:**
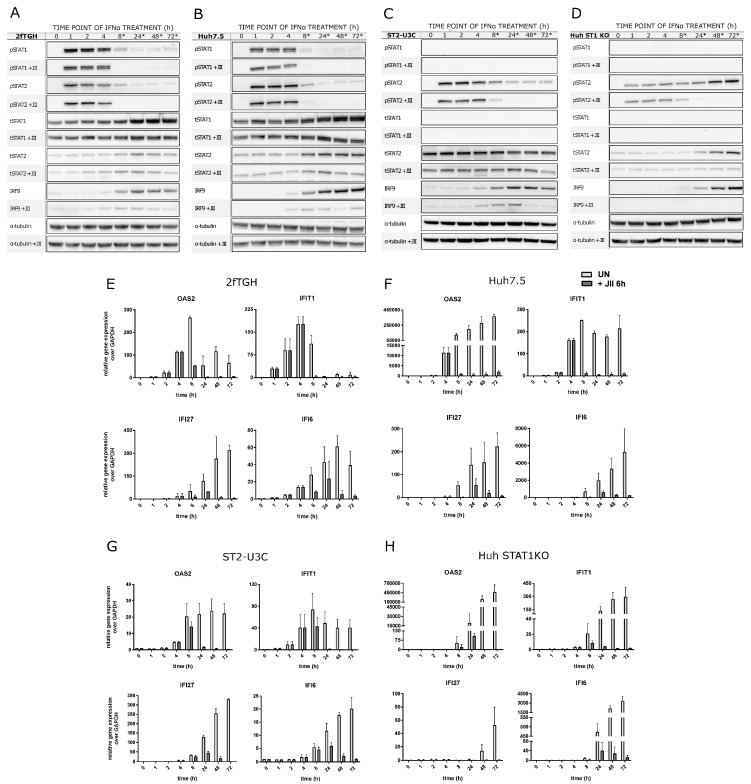
Long-term ISG expression in the WT and STAT1-KO cell lines depends on the phosphorylation of STAT2 and/or STAT1. (**A**–**D**) Immunoblot results representing the protein production and phosphorylation profiles in IFNα-treated 2fTGH (**A**), Huh7.5 (**B**), ST2-U3C (**C**) and Huh STAT1KO (**D**) cells were additionally treated or not with JAK Inhibitor I (JII) after 6 h of IFNα treatment; p—phosphorylated, t—total, *—JII treatment that overlaps with late phase of the IFNα response. (**E**–**H**) qPCR results demonstrating the expression profiles of selected ISGs (*OAS2*, *IFIT1*, *IFI27* and *IFI6*) in 2fTGH (**E**), Huh7.5 (**F**), ST2-U3C (**G**) and Huh STAT1KO (**H**) treated with IFNα and JII. Relative expression over GAPDH was estimated; *n* = 2; mean ± SEM.

First, 2fTGH and Huh7.5 cells treated with 1000 U/mL of IFNα alone for 0, 1, 2, 4, 8, 24, 48 and 72 h were compared to cells treated with IFNα alone for 0, 1, 2, 4 h, and for 8, 24, 48 and 72 h together with JII (added after 6 h of IFN treatment, at each time point, to inhibit the late IFN response phase) (Figure 4A,B). As became clear, addition of JII resulted in a complete block of STAT1 and STAT2 phosphorylation in 2fTGH at 8, 24, 48 and 72 h. Likewise, in Huh7.5, phosphorylation was inhibited at each of these time points, except at 8 h, where weak phosphorylation of STAT1 and STAT2 was still visible. In both cell types, increased production of total STAT1, STAT2 and IRF9, observed after long-term IFNα treatment, was also impaired by the addition of JAK Inhibitor I. However, expression was still detectable. This predicts the role of STAT1 and STAT2 phosphorylation in the long-term expression of the ISGF3 components and in the action of ISGF3 at later time points in 2fTGH and Huh7.5 cells. 

In a similar fashion, phosphorylation of STAT2 was significantly diminished after treatment with JII and no longer detectable in both ST2-U3C and Huh STAT1KO after 48 and 72 h of IFNα treatment (Figure 4C and Figure 4D, respectively). Like in WT cells, JII addition also significantly hampered the IFN-dependent increased production of STAT2 and IRF9, although more dramatically in Huh-STAT1KO cells than in ST2-U3C. In Huh STAT1KO, expression of IRF9 was even no longer detectable upon JII treatment. 

These observations suggest that in the STAT1-deficient cell lines, long-term expression of STAT2 and IRF9 is highly dependent on the phosphorylation of STAT2 itself. They also point to the crucial role of STAT2/IRF9 in prolonged IFNα signaling in the absence of STAT1 in ST2-U3C and Huh STAT1KO cells. 

Under the same conditions, we studied the effect of JII treatment in both WT and STAT1KO cell lines on the long-term IFNα-induced expression of a selection of ISGs, including OAS2, IFIT1, IFI27 and IFI6, via qPCR (Figure 4E–H). In 2fTGH and Huh7.5 cells, addition of JII severely decreased expression of these genes after 8 h of IFNα treatment, which correlated with a block in STAT1 and STAT2 phosphorylation (Figure 4A,B). In ST2-U3C and Huh STAT1KO, exposure to JII dramatically decreased expression of OAS2, IFIT1, IFI27 and IFI6 to nearly undetectable levels at 48 and/or 72 h of IFNα treatment (Figure 4C,D). This correlated with inhibition of STAT2 phosphorylation and a decrease in the STAT2/IRF9 levels. 

Together, these findings show that phosphorylation is a key factor in the ISGF3- or STAT2/IRF9-mediated regulation of both early and prolonged ISG expression in WT and STAT1-KO cells. At the same time, the impaired expression of unphosphorylated ISGF3 and STAT2/IRF9 components in JII treated cells cannot rule out the involvement of U-ISGF3 and U-STAT2/IRF9 under these conditions. 

### 2.6. The Role of Unphosphorylated ISGF3 Components in the Regulation of Basal ISG Expression in Cells Overexpressing STAT1, STAT2 and IRF9 

The role of unphosphorylated STATs has been proposed in the transcriptional regulation of ISGs in an IFN-independent manner [12,15]. In this respect, we observed increased basal expression of a selection of ISGs in ST2-U3C cells as compared to U3C in the absence of IFNα treatment [5]. Recently, we generated the ST2-IRF9-U3C variant, overexpressing both STAT2 and IRF9, and studied the IFNα-dependent and -independent ISG expression in comparison to ST2-U3C and U3C cells (Figure S3). As expected, IFN-induced expression of OAS2, IFI27 and IFI6 was the highest in ST2-IRF9-U3C cells, correlating with the increased levels of phosphorylated ISGF3 components (not shown). Likewise, IFN-independent expression of these genes was also significantly higher in ST2-IRF9-U3C cells, which could point to the regulatory role of U-STAT2/IRF9 in basal ISG expression (Figure S3). Subsequently, we generated the U3C-based cell line ST1-ST2-IRF9-U3C to examine the effect of overexpressing all the ISGF3 components STAT1, STAT2 and IRF9 and the possible role of U-ISGF3 in mediating basal ISG expression. 

**Figure 5:**
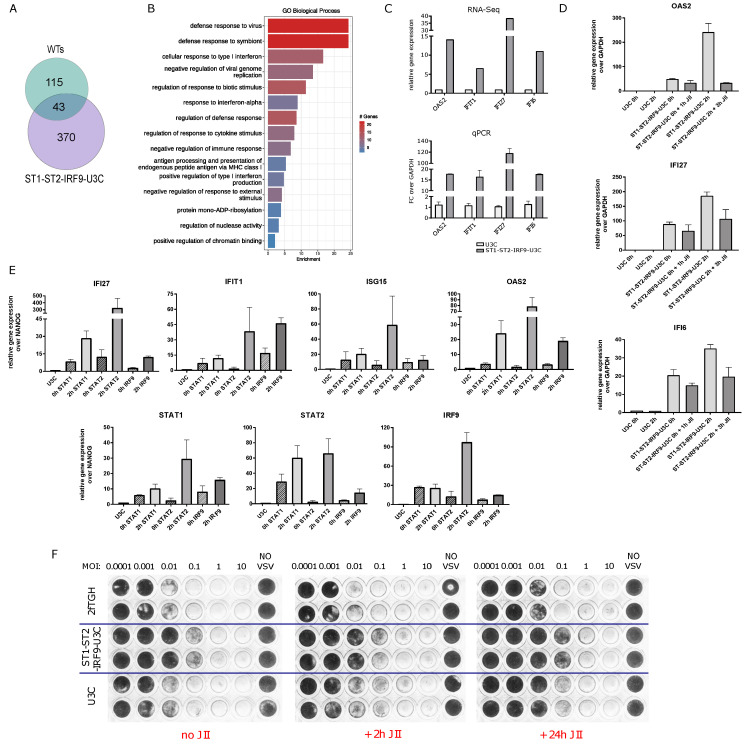
Overexpression of STAT1, STAT2 and IRF9 is sufficient to drive transcriptional responses and viral protection in the absence of IFNα treatment. (**A**) Venn diagram based on the RNA-Seq results demonstrating 43 commonly upregulated ISGs in ST1-ST2-IRF9-U3C vs. WTs (2fTGH vs. Huh7.5). (**B**) Gene Ontology terms enrichment analysis of the 43 commonly upregulated genes in ST1-ST2-IRF9-U3C and WT cells, significant enrichment considered with FDR < 0.05. (**C**) Representative ISG expression levels of *OAS2*, *IFIT1*, *IFI27* and *IFI6*, in untreated ST1-ST2-IRF9-U3C vs. untreated U3C based on RNA-Seq results (upper panel, log_2_FC > 0.5; padj < 0.05) validated by qPCR (lower panel, *n* = 2; mean ± SEM). (**D**) Phosphorylation independence of ISG expression (of *OAS2*, *IFI27* and *IFI6*) studied via treatment with IFNα and JAK Inhibitor I (JII) demonstrated using qPCR, *n* = 2; mean ± SEM; U3C used as control. (**E**) Chromatin interactions of STAT1, STAT2 and IRF9 with regulatory regions of selected ISGs (including *IFI27*, *IFIT1*, *ISG15*, *OAS2*, *STAT1*, *STAT2* and *IRF9*) in ST1-ST2-IRF9-U3C examined via ChIP-PCR, *n* = 2; mean ± SEM; relative binding was calculated over NANOG and U3C Ct values were used as a negative control; cells were treated for 2 h or left untreated. (**F**) Ability to combat viral infection of JII-treated ST1-ST2-IRF9-U3C cells (and U3C and 2fTGH as a control) in the absence of IFNα, as examined using an antiviral assay; JII treatment for 0, 4 or 24 h before 24 h VSV infection.

To compare the genome-wide basal gene expression in ST1-ST2-IRF9-U3C and U3C, we performed RNA-Seq on RNA from three independent repeats. Differential gene expression analysis was performed and upregulated genes were selected based on the cut-off of log_2_FC > 1 and padj < 0.05 (see Material and Methods). As such, 413 genes were identified in ST1-ST2-IRF9-U3C cells with increased basal expression (Figure 5A). After comparing these genes with the 158 common IFNα-induced genes in WT cells (Table S2), 43 genes were found in common (Figure 5A). GO term enrichment analysis revealed significant enrichment in biological terms connected to Type-I IFN signaling as well as broadly defined antiviral responses, and it recognized these genes among the core subset of antiviral ISGs (Figure 5B). Among the 43 genes, the above characterized pre-selected 11 ISRE-containing antiviral ISGs could be observed (Figure 1G,H), as well as many additional known ISRE-containing ISGs, including DDX60, IFITM1 and IFITM 3 as well as BST2 (Table S2). Indeed, as compared to U3C, ST1-ST2-IRF9-U3C cells exhibited higher basal expression of OAS2, IFIT1, IFI27 and IFI6 (Figure 5C). Pre-treatment with JII for 1 h did not significantly lower the basal expression of a number of these genes (including OAS2, IFI27 and IFI6) in untreated ST1-ST2-IRF9-U3C cells (Figure 5D). Treatment with IFNα for 2 h increased the expression of OAS2, IFI27 and IFI6, returning to basal levels upon JII pre-treatment. This is in agreement with the role of phosphorylation in IFN-I-activated transcriptional responses and provides further evidence of a phosphorylation-independent mechanism involved in the regulation of basal ISG expression in ST1-ST2-IRF9-U3C cells. 

Therefore, we characterized the chromatin interactions of the ISGF3 components to the regulatory regions of IFIT1, ISG15, OAS2, IFI27, STAT1, STAT2 and IRF9 via ChIP-PCR (Figure 5E). Chromatin was isolated from untreated and IFNα-treated ST1-ST2-IRF9-U3C cells, as well as untreated U3C, using antibodies against STAT1, STAT2, and IRF9. Increased binding of all three ISGF3 components could be detected at the ISRE sites present in the promoter of these genes in untreated ST1-ST2-IRF9-U3C cells as compared to U3C. As expected, binding was even further increased in IFN-treated ST1-ST2-IRF9-U3C cells (Figure 5E). 

Next, we studied the ability of ST1-ST2-IRF9-U3C cells to combat viral infection in the absence of IFN treatment. In comparison to U3C and 2fTGH, cells were infected with serial 10-fold dilutions of Vesicular Stomatitis Indiana (VSV) virus starting from MOI = 10 (Figure 5F). U3C, as well as 2fTGH cells, were not able to fight with the virus at an MOI more than 0.001. Oppositely, ST1-ST2-IRF9-U3C cells were protected 10–100× more against VSV infection (up to MOI = 0.1). Pre-treatment with JAK Inhibitor I for 2 or 24 h did not affect this antiviral potential of ST1-ST2-IRF9-U3C cells (compare Figure 5F: no JII vs. JII 2 h vs. JII 24 h). 

### 2.7. The Role of Unphosphorylated ISGF3 Components in Prolonged IFNα Signaling in Cells Overexpressing STAT1, STAT2 and IRF9 

**Figure 6:**
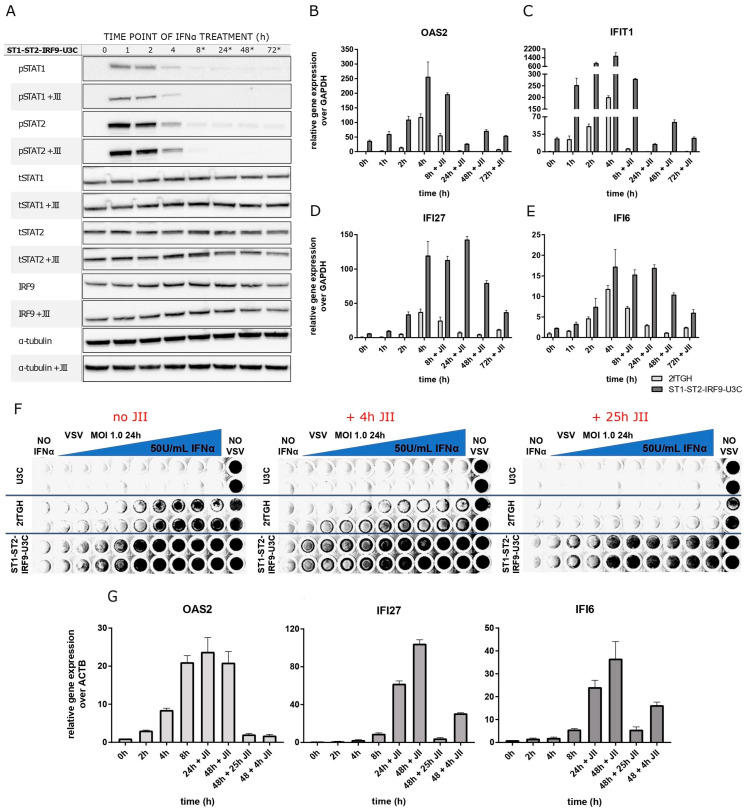
Long-term ISG expression upon abundance of STAT1, STAT2 and IRF9 depends on the phosphorylation process. (**A**) Immunoblot results representing the protein production and phosphorylation profiles in the IFNα-treated ST1-ST2-IRF9-U3C additionally treated or not with JAK Inhibitor I (JII) for 6 h; *—JII treatment overlaps with late phase of IFNα response. (**B**–**E**) qPCR results demonstrating the expression profile of selected ISGs (*OAS2*, *IFIT1*, *IFI27* and *IFI6*) in ST1-ST2-IRF9-U3C and 2fTGH treated with IFNα and JII. Relative expression over GAPDH was estimated; *n* = 2; mean ± SEM. (**F**) Ability to combat viral infection of the IFNα- and JII-treated ST1-ST2-IRF9-U3C cells (and U3C and 2fTGH as a control) examined using an antiviral assay; JII added 0, 4 or 25 h before twenty-four-hour IFNα treatment, followed by VSV infection for another 24 h, (**G**) validated via examination of selected ISG expression profiles (including *OAS2*, *IFI27* and *IFI6*) using qPCR, *n* = 2; mean ± SEM.

Finally, we examined the effect of overexpressing the ISGF3 components STAT1, STAT2 and IRF9 and the possible role of U-ISGF3 in mediating IFNα-dependent long-term ISG expression in ST1-ST2-IRF9-U3C cells. Growing the cells in the absence or presence of IFNα revealed that phosphorylation of STAT1 and STAT2 was absent in the controls but highly induced upon IFNα treatment with an early, transient increase between 1 and 4 h, after which it diminished to still detectable levels at 72 h (Figure 6A). As STAT1, STAT2 and IRF9 are overexpressed in these cells, their protein expression was not affected by IFN treatment. 

Addition of JII resulted in a severe drop in STAT1 and STAT2 phosphorylation at 8, 24, 48 and 72 h. On the other hand, no effect was seen on the native levels of STAT1, STAT2 as well as IRF9. Comparison of IFN-stimulated 2fTGH and ST1-ST2-IRF9-U3C cells, treated with JII under the same conditions, revealed a significant difference in the gene expression levels of *IFIT1*, *OAS2*, *IFI27* and *IFI6* after long-term IFNα and JII treatment (Figure 6B–E). In contrast to 2fTGH, expression of these ISGs was still detectable in the presence of JII, even after 72 h. Moreover, higher basal expression of *IFIT1*, *OAS2*, *IFI27* and *IFI6* could be detected in ST1-ST2-IRF9-U3C cells as compared to 2fTGH, and in the presence of IFNα and JII, their expression never dropped below the basal level observed in untreated ST1-ST2-IRF9-U3C cells. 

Next, we compared the ability of 2fTGH and ST1-ST2-IRF9-U3C cells to generate an IFN-activated antiviral response in the presence or absence of JII (Figure 6F). Thus, cells were treated with a two-fold serial dilution of IFNα for 24 and then infected with VSV for another 24 h. During this infection time, cells were treated with JII for 4 h or 25 h. As a control, U3C cells were used and treated similarly. The results clearly showed that in 2fTGH cells, antiviral activity is strongly dependent on phosphorylation. Consequently, addition of JAK Inhibitor I rendered these cells more sensitive to VSV infection. Pre-treatment of 2fTGH cells with IFNα could not effectively protect them from virus-dependent lysis. Importantly, 4 h of stimulation with JII only partially impaired IFNα-mediated 2fTGH cell viral protection. However, addition of JII for 25 h completely blocked viral protection. On the other hand, in ST1-ST2-IRF9-U3C cells, blocking phosphorylation did not have a major effect on IFN-induced antiviral activity. The qPCR results under these conditions indeed confirmed that OAS2, IFI27 and IFI6 were still significantly expressed at later time points and never dropped below basal levels, being sufficient to protect cells from lysis by VSV (Figure 6G).

## 3. Discussion

In accordance with the general paradigm of IFN-I signaling, a robust and transient phosphorylation pattern of STAT1 and STAT2 is followed by a similar ISG expression profile that decreases over time. Conversely, recent studies revealed the increasing complexity of this response, with more prolonged ISG expression patterns that were shown to rely on sustained expression of the components of ISGF3 as part of a positive feedback loop [2,12,13,14]. In this context, Cheon et al. [12] postulated a novel model of how antiviral effects are prolonged after IFNβ exposure. In the early response phase, phosphorylation of STAT1 and STAT2 correlated with formation of ISGF3 and transcriptional regulation of many ISGs, including STAT1, STAT2, and IRF9. A drop in STAT phosphorylation during the course of a few hours corresponded with a parallel decrease in expression of a subset of early ISGF3 target genes (e.g., *IRF1*, *ADAR*, and *MYD88*). In contrast, at later times, after IFN stimulation, high levels of IRF9 together with U-STAT1 and U-STAT2 proteins increased formation of U-ISGF3 and prolonged expression of a subset of U-ISGs (*IFI27*, *OAS2*, *MX1*, *BST2*, *IFIT1*, and *IFIT3*; see also Sung et al. [13]), which were previously found to be induced by U-STAT1 [17]. Likewise, Sung et al. [13] observed that the level of U-ISGF3, but not tyrosine phosphorylated STAT1, was significantly elevated in response to IFN-λ and IFN-β during chronic HCV infection. Subsequently, U-ISGF3 prolonged the expression of U-ISGs and restricted HCV chronic replication. 

Using RNA-Seq and ChIP-Seq, we further assessed the genome-wide comparative role of these ISGF3 and ISGF3-like complexes in connection to constitutive and long-term IFNα-treated ISG expression and antiviral activity. First, we identified a group of ISRE-containing ISGs that were commonly upregulated in IFNα-treated WT and STAT1-KO cells. Thus, in 2fTGH and Huh7.5 WT cells, IFNα-inducible transcription and antiviral activity relied on the recruitment of the ISGF3 components STAT1, STAT2 and IRF9 in a phosphorylation- and time-dependent manner. Indeed, in these cells, the phosphorylation and chromatin binding of STAT1 and STAT2 was still clearly visible after 72 h. Moreover, no shift could be detected from binding of phosphorylated STATs to U-STATs at later time points, which is in contrast to the phosphorylation-independent model proposed by Cheon et al. [12]. Along the same lines, our data also disagree with the existence of ISGs and U-ISGs, as suggested by Cheon et al. [12] and Sung et al. [13]. Instead, the U-ISG expression profiles followed a similar pattern as the common IFNα-inducible ISGs. More importantly, the binding profiles of the different antibodies to these genes resembled the commonly IFNα-inducible ISG peak distribution, with STAT1, STAT2, pSTAT1, pSTAT2 and IRF9 binding peaks still detectable after 72 h. Although for the majority of the pre-selected ISGs in our study (*IFIT1*, *IFIT2*, *IFIT3*, *ISG15*, *OAS1*, *OAS2*, *STAT1*, *STAT2* and *IRF9*) involvement of the ISRE sequence in transcription was shown, additional functional analysis should be carried out to prove this for the remaining pre-selected ISGs and U-ISGs.

Together with our recently published data [2,14], these results are in line with the dominant role of classical ISGF3, and not U-ISGF3, in the regulation of early as well as prolonged ISG expression and viral protection in 2fTGH and Huh7.5 cells. More importantly, they disagree with the model proposed by Cheon et al. [12] and Sung et al. [13], in which U-ISGF3 may switch with ISGF3 to drive prolonged expression of U-ISGs in response to IFN-I.

The importance of the STAT2/IRF9 complex in long-term IFN responses under conditions of STAT1 deficiency has been addressed in different studies. For example, Lou et al. [6] reported that STAT2 together with IRF9 can effectively drive the transcription of the RIG-G gene via their functional interaction, even without tyrosine phosphorylation of STAT2. We showed previously that in the absence of STAT1, STAT2 is capable of forming homodimers when phosphorylated in response to IFN-I [4]. Together with IRF9, these STAT2 homodimers formed STAT2/IRF9 that activated transcription of ISRE-containing genes in response to IFNα [4]. In a more genome-wide setting, this STAT2/IRF9 complex triggered the expression of a similar subset of ISGs as ISGF3, although with a more prolonged expression profile [1,5,6]. As a consequence, STAT2/IRF9 was able to initiate an antiviral response upon EMCV and VSV, offering additional proof of the functional overlap between STAT2/IRF9 and ISGF3. 

Our data here additionally show that IFN-I-induced expression of these commonly induced ISRE-containing ISGs was delayed in ST2-U3C and Huh-STAT1KO cells lacking STAT1 (as compared to WT cells) and associated with DNA binding of phosphorylated STAT2/IRF9. Also, no shift could be detected from binding of phosphorylated STAT2 to U-STAT2 at later time points, agreeing with the observation that the U-ISG expression and binding profiles in ST2-U3C and Huh-STAT1KO cells displayed a similar pattern as identified for the common IFNα-inducible ISGs. Previous studies by Blaszczyk et al. [5] suggested that, in the absence of STAT1, a certain threshold amount of STAT2 and IRF9 must be reached to allow STAT2 phosphorylation and STAT2/IRF9-mediated transcription. With the low to undetectable IRF9 levels in untreated ST2-U3C and Huh ST1KO cells, it takes a certain time for IFN treatment to synthesize enough IRF9 and, together with the already available STAT2, reach this threshold. Moreover, subtle differences between ST2-U3C and Huh ST1KO cells in these threshold levels could potentially explain the differences in their response to IFN*α*. The lower DNA affinity of the STAT2/IRF9 complex as compared with ISGF3, on the other hand, requires abundance of STAT2 and IRF9 protein and correlates with the delayed and prolonged nature of its IFN*α*-mediated activity [5]. In combination with phosphorylation inhibition experiments using the JAK inhibitor JI1, our findings highly suggest that in analogy to ISGF3, phosphorylation is also a key factor in the STAT2/IRF9-mediated DNA binding and regulation of prolonged ISG expression in STAT1-KO cells. The data also offer further proof of the previous observation that STAT2/IRF9 can take over the role of ISGF3 and generate an antiviral response in the absence of STAT1 [4,5,9]. This is in agreement with Yamauchi (2016), who observed in HCV-infected Huh-7.5 human hepatoma cells that IFN-α activated transcription of ISRE genes and inhibited HCV replication through a STAT2-dependent but STAT1-independent pathway. In contrast, IFN-λ induced ISG expression and inhibited HCV replication exclusively through a STAT1- and STAT2-dependent pathway. Additional in vivo evidence was provided by Abdul-Sater [7] and Perry [8] of the existence of a STAT2/IRF9-dependent, STAT1-independent host defense mechanism against Dengue virus and Legionella pneumophila, respectively. These studies confirm that IFN-I is able to drive the formation of STAT2/IRF9, which interacts with DNA and regulates expression of a subset of ISRE-containing ISGs, offering a back-up response against viral infection. Under these conditions, our data are also not in line with a ‘switch’ model from STAT2/IRF9 to U-STAT2/IRF9, as proposed for ISGF3 by Cheon et al. [12] and Sung et al. [13], to drive prolonged expression of U-ISGs in response to IFN-I.

The accumulation of U-STAT1, U-STAT2 and IRF9 in time, marking the positive feedback regulation of the ISGF3 components observed in 2fTGH and Huh7.5 cells in response to IFNα, raised the possibility of an additional role of U-ISGF3 in mediating prolonged IFN Type-I signaling. Similarly, in STAT1KO cells, accumulation of IRF9 and U-STAT2 was observed (pre-dominantly at later time points) and pointed to a potential role of U-STAT2/IRF9 in long-term IFN Type-I signaling in ST2-U3C and Huh-STAT1KO cells. Moreover, the impaired expression of unphosphorylated ISGF3 and STAT2/IRF9 components in JII treated cells proves the importance of phosphorylated ISGF3 and STAT2/IRF9 complexes but cannot rule out the involvement of U-ISGF3 and U-STAT2/IRF9 under these conditions. To address this issue, we generated the U3C-based cell line ST1-ST2-IRF9-U3C to examine the effect of overexpressing the ISGF3 components STAT1, STAT2 and IRF9 and the possible role of U-ISGF3 in mediating basal ISG expression. Indeed, comparative experiments in U3C (STAT1-KO) cells overexpressing all the ISGF3 components (ST1-ST2-IRF9-U3C) revealed increased expression of ISRE genes and antiviral activity independent of phosphorylation and IFN treatment. Moreover, binding of all three ISGF3 components could be detected at the ISRE sites present in the promoter of pre-selected ISGs in untreated ST1-ST2-IRF9-U3C cells as compared to U3C. 

Our data agree with a model proposed by Wang et al. [15], in which constitutive expression of ISGs in immortalized cell lines, primary intestinal and liver organoids, and liver tissues was shown to depend on U-ISGF3. Moreover, their analysis of a pre-existing ChIP-Seq dataset (GSE31477) [15] claimed that STAT1 specifically bound, although with very low affinity, to the promoters of ISGs even in the absence of IFNs. In contrast, analysis of our ChIP-Seq datasets here on untreated 2fTGH, Huh7.5, ST2U3C and Huh7.5 STAT1KO provide no proof of basal binding of U-STAT1, U-STAT2 and IRF9. Likewise, no basal DNA binding of unphosphorylated ISGF3 components could be detected in human THP1 cells as opposed to mouse macrophages under physiological conditions [16]. However, ISRE binding of STAT1, STAT2 and IRF9 in untreated ST1-ST2-IRF9-U3C cells, overexpressing all the ISGF3 components, provided clear proof of a role of U-ISGF3 in basal ISG transcription. Likewise, in Wang’s study, simultaneous overexpression of all the ISGF3 components, but not any single factor, induced the expression of ISGs and inhibited viral replication; however, no phosphorylated STAT1 and STAT2 were detected. Moreover, a phosphorylation-deficient STAT1 mutant was comparable to the wild-type protein in mediating the IFN-independent expression of ISGs and antiviral activity. Based on this, it is tempting to speculate that a certain threshold of STAT1, STAT2 and especially IRF9 expression and levels of U-ISGF3 have to be reached to be able to trigger basal ISG transcription. However, carefully designed single-cell experiments would be needed to confirm such a hypothesis. A similar mechanism could account for a role of U-STAT2/IRF9 in U3C cells overexpressing STAT2 or STAT2+IRF9, displaying increased basal ISG expression as compared to U3C cells. In this respect, Platanitis [16] showed that the preformed STAT2/IRF9 complexes control basal ISG expression in murine macrophages. So far, this has not been shown in human cells. It would be interesting to see if the differences in basal expression levels of the ISGF3 components in mouse and human cells could account for these contrasting observations. At the same time, overexpression of the ISGF3 components STAT1, STAT2 and IRF9 provided conditions for the possible role of U-ISGF3 in mediating IFNα-dependent long-term ISG expression in ST1-ST2-IRF9-U3C cells. Indeed, in ST1-ST2-IRF9-U3C cells, blocking phosphorylation did not have a major effect on IFN-induced antiviral activity, whereas ISG transcription never dropped below basal levels, being sufficient to protect cells from lysis by VSV. Based on this, it is tempting to suggest that in addition to the dominant role of classical ISGF3 in the regulation of early as well as prolonged ISG expression and viral protection, U-ISGF3 (and possibly U-STAT2/IRF9) could have additional involvement. This observation is consistent with studies published by Majoros et al. [18], which show a potential contribution of Y701-unphosphorylated STAT1 to innate antibacterial immunity. 

Collectively, our data are in line with the dominant role of classical ISGF3 and STAT2/IRF9 in the IFN-dependent DNA binding and regulation of early as well as prolonged ISG expression and viral protection. Our data disagree with a model in which U-ISGF3 or U-STAT2/IRF9 may switch with ISGF3 or STAT2/IRF9 to drive prolonged expression of U-ISGs in response to IFN-I. However, they are in favor of an additional, threshold-dependent role of U-ISFG3, and possibly U-STAT2/IRF9, in the regulation of constitutive and possibly IFN-dependent ISG expression. As a consequence, together with phosphorylated ISGF3 and STAT2/IRF9 complexes, U-ISGF3 and U-STAT2/IRF9 could be instrumental in IFN-dependent and -independent ISG transcription and antiviral activity. 

## 4. Materials and Methods

### 4.1. Cell Lines

The human fibrosarcoma 2fTGH cell line and the STAT1-deficient U3C cell line were kind gifts from Prof. Sandra Pellegrini (Institute Pasteur, Paris, France). The U3C is a STAT1-deficient clone derived from 2fTGH using a high-frequency mutagenesis screen and belongs to the same complementation group as U3A (Prof. Sandra Pellegrini: personal communication; see Blaszczyk et al., 2015 [5] and McKendry 1991 [19]). The U3C cell lines stably overexpressing combinations of ISGF3-components (STAT2-U3C, STAT2-IRF9-U3C, STAT1-STAT2-IRF9-U3C) were generated in our laboratory using MigR1 vectors containing the genes of interests and pcDNA6/TR (blasticidin resistance; ThermoFisher Scientific, Waltham, MA, USA, V102520), which were transfected with Xtreme HP reagent (Roche, Basel, Switzerland, 6366236001).

The human hepatocellular carcinoma cell lines Huh7.5 WT and Huh STAT1 K.O., which were derived from Huh7.5 using the CRISPR/Cas9 system [9,14], were a kind gift from Prof. Kiyonao Sada (Department of Genome Science and Microbiology, University of Fukui, Fukui, Japan).

#### Cell Culture and Treatment

All the cell lines were cultured in DMEM (IITD PAN Wrocław, 11) supplemented with 10% fetal bovine serum (FBS) (ThermoFisher Scientific, 10500-064), 2 mM L-glutamine (BioWest, Lakewood Ranch, FL, USA, X0550) and 100 IU/mL penicillin/100 µg/mL streptomycin/250 ng/mL amphotericin B (Sigma-Aldrich, St. Louis, MO, USA, A5955) and, in the case of Huh7.5 WT and Huh7.5 STAT1 K.O., also with 1% MEM NEAA (ThermoFisher Scientific, 11140-035). Both media are referred to later in the manuscript as full culture media. The cells were starved with medium containing 1% FBS concentration and treated with IFNα (1000 U/mL, Merck-Milipore, Burlington, MA, USA, IF007) up to 72 h and with JAK Inhibitor I (JII, Pyronide 6, 5 μM, Bio-techne, Minneapolis, MN, USA, 6577/10) up to 25 h, depending on the experiment. For clarifying the presented RNA-Seq and ChIP-Seq data, on the heatmaps and boxplots in Figure 1 and Figure 3 we showed unified timepoints. For the ST2STAT1-KO cell lines, we used later time points as in these cell types the response is prolonged. Unless mentioned differently, all the experiments were performed as two independent biological repeats (*n* = 2).

### 4.2. Western Blotting

Protein isolation, quantification and immunoblotting were performed as described before by Piaszyk-Borychowska [20] with the use of the primary antibodies anti-pSTAT1 (CST, 7649, D4A7, 1:200), -pSTAT2 (CST, 88410, D3P2P, 1:500), -tSTAT1 (CST, 14994, D1K9Y, 1:500), -STAT2 (CST, 72604, D9J7L, 1:500), -IRF9 (CST, 76684, D2T8M, 1:400 in case of 2fTGH and U3C-based cells and 1:300 in case of Huh cells) as well as -α-tubulin (Abcam, Waltham, MA, USA, AB52866, 1:2000) and the secondary HRP-conjugated goat anti-rabbit antibodies (Sigma-Aldrich, A9169, 1:20,000).

### 4.3. RNA Isolation and qPCR

The total RNA was extracted using the GeneMATRIX Universal RNA Purification Kit (EurX, Gdansk, Poland, E3598-02) according to the protocol provided by the manufacturer. A total of 500 ng of purified total RNA was then reverse-transcribed using ThermoFisher Scientific reagents (K1622). Quantification of transcripts was performed via qPCR with Maxima SYBR Green/ROX qPCR Master Mix (K0223, TFS) using the CFX Connect Thermal Cycler System (Bio-Rad Laboratories, Herciles, CA, USA). The target gene levels were normalized to glyceraldehyde-3-phosphate dehydrogenase (GAPDH) and quantified as described by Willems et al. [21]. The sequences of the primers used are shown in Table S3. The relative gene expression results, if not indicated differently, are presented as the mean +/− SEM for two independent biological repeats. The graphs were prepared using GraphPad Prism 7.01 [22].

### 4.4. RNA-Seq Library Preparation and Sequencing

RNA was isolated from cells treated with IFNα up to 72 h, depending on the experiment type, and quantified using a Qubit RNA BR assay kit (Q10210 ThermoFisher Scientific), and the quality was assessed via the Agilent 2100 Bioanalyzer using the RNA 6000 Nano kit (5067-1511, Agilent Technologies, Santa Clara, CA, USA) according to the protocols provided by manufacturers. RNA degradation was assessed by means of RIN (RNA integrity number) and samples with RIN higher than 9 were then used for further analysis. RNA libraries were prepared in three biological repeats from 1 μg of total RNA using NEBNext^®^ Ultra™ or Ultra™ II RNA Library Prep Kit for Illumina^®^ (New England Biolabs, NEB, Ipswitch, CA, USA) together with NEBNext Poly(A) mRNA Magnetic Isolation Module (NEB) and NEBNext^®^ Multiplex Oligos for Illumina^®^ (NEB) according to the manufacturer’s protocol. The quality and fragment distribution of the prepared libraries were estimated using the Agilent High Sensitivity DNA kit (5067-4626, Agilent Technologies) and the quantity was assessed using the Qubit dsDNA HS assay kit (Q32851, ThermoFisher Scientific). Sequencing (HighOutput SR75, v2 chemistry) was performed on the NextSeq500 (Illumina, San Diego, CA, USA) in Lexogen, BioCenter in Vienna, Austria.

### 4.5. RNA-Seq Data Analysis

For ST1-ST2-IRF9-U3C, Salmon v1.10.2 [23] has been used to count the transcript-level abundance estimates with a gencode.v43.basic.annotation GTF file. For the rest of the cell lines, Fastq files were aligned using STAR v2.7.3a [24] against the Homo_sapiens.GRCh38.dna.primary_assembly genome build (release-100). The gene counts (reads aligned to each gene of each sample) were generated using FeatureCounts v1.6.2 with default parameters [25]. Quality control assessments of the raw and mapped read counts were carried out using FastQC [26] and the reports were combined with MultiQC [27]. Genes with low counts (below 10 in any time point) were considered as ‘non-expressed’ and filtered out for downstream analysis. 

#### 4.5.1. Differential Gene Expression Analysis (DEG)

The counts were normalized and DEG analysis was performed using the DESeq2 v1.30.1 package [28] in R v4.0.3 software [29]. The Wald test (for ST1-ST2-IRF9-U3C and U3C) or likelihood ratio test (LRT; for the rest of the cell lines) was used to identify genes that respond to IFNα treatment over time. The relationships between the replicates and time of treatment were assessed via a principal component analysis (PCA) plot generated using DESeq2. The false discovery rate (FDR)-adjusted q-values (5% threshold) were calculated via the Benjamini–Hochberg procedure. The log_2_FC (fold change) was also calculated for each gene. Genes with adjusted *p*-values (padj) less than 0.05 and log_2_FC > 0.5 (for ST2-U3C and ST1-ST2-IRF9-U3C) or log_2_FC > 1 (for the rest of the cell lines) were considered as DEGs.

#### 4.5.2. Heatmap Generation

The heatmap visualizing the transcriptional response to IFNα stimulation was prepared using the pheatmap v1.0.12 [30] and ComplexHeatmap v2.10.0 [31] packages (R). For selected genes, the normalized counts obtained from DESeq2 were extracted and subjected to hierarchical clustering (by row) with the default clustering method: complete, Euclidean distance. For plotting, row scaling with Z-scores was performed. The color scale (from blue to red, which represents low and high normalized intensity, respectively) indicates the expression change over time for each sample compared to the expression of the non-stimulated control.

#### 4.5.3. Gene Ontology Term Enrichment Analysis

The GO term enrichment analysis was performed using the R package clusterProfiler 4.6.0 [32,33] with following settings: (1) all the differentially expressed genes from 2fTGH (14 920) as background, (2) *p*-value cutoff 0.01 and q-value cutoff 0.05 on enrichment tests, (3) *p*-values adjustment method Benjamini and Hochberg, and (4) sub-ontology Biological Process (BP). To reduce the redundancy of the enriched GO terms, the ‘simplify’ function was applied, with default parameters and cutoff 0.6. A total of 15 terms with the highest statistical significance were used for visualization as bar plots (ggplot2 3.4.2) [34]. Enrichment was defined as −log_10_(adjusted *p*-value).

#### 4.5.4. Selection of Commonly Upregulated Genes

To select the commonly upregulated genes, Venn diagrams were prepared using jvenn software [35] and Inkscape [36].

### 4.6. Chromatin Immunoprecipitation (ChIP) and Sequencing (ChIP-Seq)

The ChIP experiments were prepared with chromatin isolated from cells treated with IFNα for 2, 24 and 72 h or left untreated. ChIP was performed as previously described [5] using the antibodies listed in the Western blotting paragraph. The immunoprecipitated chromatin concentration was measured using a Qubit dsDNA HS assay kit (Q32851, ThermoFisher Scientific). Chromatin was diluted 13× and quantified using the qPCR method and primers listed in Supplementary Table S4. The relative binding was calculated over NANOG and the U3C Ct values were used as a negative control.

### 4.7. ChIP-Seq Data Analysis

For Huh7.5 and Huh ST1 K.O., the ChIP-Seq data were analyzed according to the ENCODE Transcription Factor processing pipeline (https://github.com/ENCODE-DCC/chip-seq-pipeline2) with default parameters [37], as described previously [14]. The analysis of the rest of the cell lines was carried out as follows. After quality assessment of the raw reads with FastQC, the alignment with the GRCh38.106 human genome assembly was performed with Bowtie2 v2.4.1 [38] and BAM files were created and sorted using SAMTools v1.6 [39]. PCR duplicates were removed using BamUtil v1.0.15 [40]. The read enrichments (peaks) of the transcription factors were predicted using MACS v2.2.6 [41]. During the secondary analysis, artefacts were determined and removed based on the blacklisted genes list from the ENCODE project [37] using BEDTools v2.30.0 [42].

To annotate the location of a given peak in terms of the important genomic features and to associate peaks with nearby genes, the annotatePeaks.pl program from HOMER v4.11 was used [43].

#### 4.7.1. Visualization in the Integrative Genomics Viewer

To visualize the ChIP-Seq data in the Integrative Genomics Viewer (IGV), BAM files obtained using the Bowtie2 aligner were converted with bamCoverage (deepTools2 v3.5.0) [44] to the bigwig format. IGV snapshots were taken in order to present them in the figures [45].

#### 4.7.2. Binding Profiles

In order to generate STAT1, STAT2, IRF9, pSTAT1 and pSTAT2 binding profiles upon IFNα treatment by time point, the matrices that represent the binding signal over selected genomic intervals have been quantified prepared as follows. For each treatment, the *p*-value signal tracks from pooled replicates per each time points and the bed file with peaks annotated to promoters of selected 63 (commonly upregulated genes in wild-type vs. STAT1 knock-out cell lines) genes were used. The signals were computed across 4 kb region centered on the ChIP-Seq peak summits with the computeMatrix function from deepTools v3.5.0 (reference-point TSS, upstream region 3000 bp, downstream region 1000 bp) [44]. Further steps were performed with the profileplyr_1.10.0 package [46]. First, k-means clustering of the signal across the genomic intervals of gene promoters was performed with the pheatmap package [30]. The mean range signal for each cluster was subsequently visualized as boxplots with ggplot2 [34]. Statisctical analyses have been performed using the *t*-test (ggpubr package).

#### 4.7.3. Binding Site Motifs Identification

Peaks annotated for the 63 commonly upregulated genes in wild-type vs. STAT1 knock-out cell lines were used to identify the enriched transcription-factor-binding motifs using Hypergeometric Optimization of Motif EnRichment (HOMER) v. 4.9.1 [43]. The matrices selected in Sekrecka et al. [14] for the binding elements annotation (4 for GAS and 3 for ISRE), shown in Figure S1, were applied for the binding site annotation using the annotatePeaks.pl program (HOMER). The motif logos were generated using the universal motif package v1.12.1 [47].

### 4.8. Deposited Sequencing Data

RNA sequencing and/or ChIP sequencing data for 2fTGH, ST2-U3C, Huh STAT1KO, ST1-ST2-IRF9-U3C and U3C are under submission at NCBI GEO DataSets. For Huh7.5, RNA-Seq and ChIP-Seq data have been submitted to GEO under the accession numbers SuperSeries GSE222668 and GSE247728.

### 4.9. Antiviral Assay

Two different schemes were used for the antiviral assay. One, classical, was performed as described before [48,49] on cells pre-treated with or without 2-fold serial dilutions of IFNα, starting from 50 U/mL, for 24 h. The second was performed on untreated cells. Both procedures, besides the IFN treatment, were performed in the same way: Vesicular Stomatitis Indiana virus (VSV) at a multiplicity of infection (MOI) of 1.0 was added to the cells using serum-free DMEM. Twenty-four hours post-infection, the medium was removed and the cells were fixed with a 10% formaldehyde solution for 20 min at room temperature. After fixation, the cells were visualized via crystal violet staining.

## Data Availability

Data are available under the accession number SuperSeries GSE247728.

## Supplementary Materials

The following supporting information can be downloaded at https://www.mdpi.com/article/10.3390/ijms242417635/s1.
