# Supplementary material for: ISGF3 and STAT2/IRF9 Control Basal and IFN-Induced Transcription through Genome-Wide Binding of Phosphorylated and Unphosphorylated Complexes to Common ISRE-Containing ISGs
Source: Int J Mol Sci. 2023 Dec 18;24(24):17635. doi: 10.3390/ijms242417635 (PMC10743977; doi:10.3390/ijms242417635)
Supplement: Supplementary file 1 [file ijms-24-17635-s001.zip › ijms-2694904-supplementary.pdf]

Supplementary Figure S1

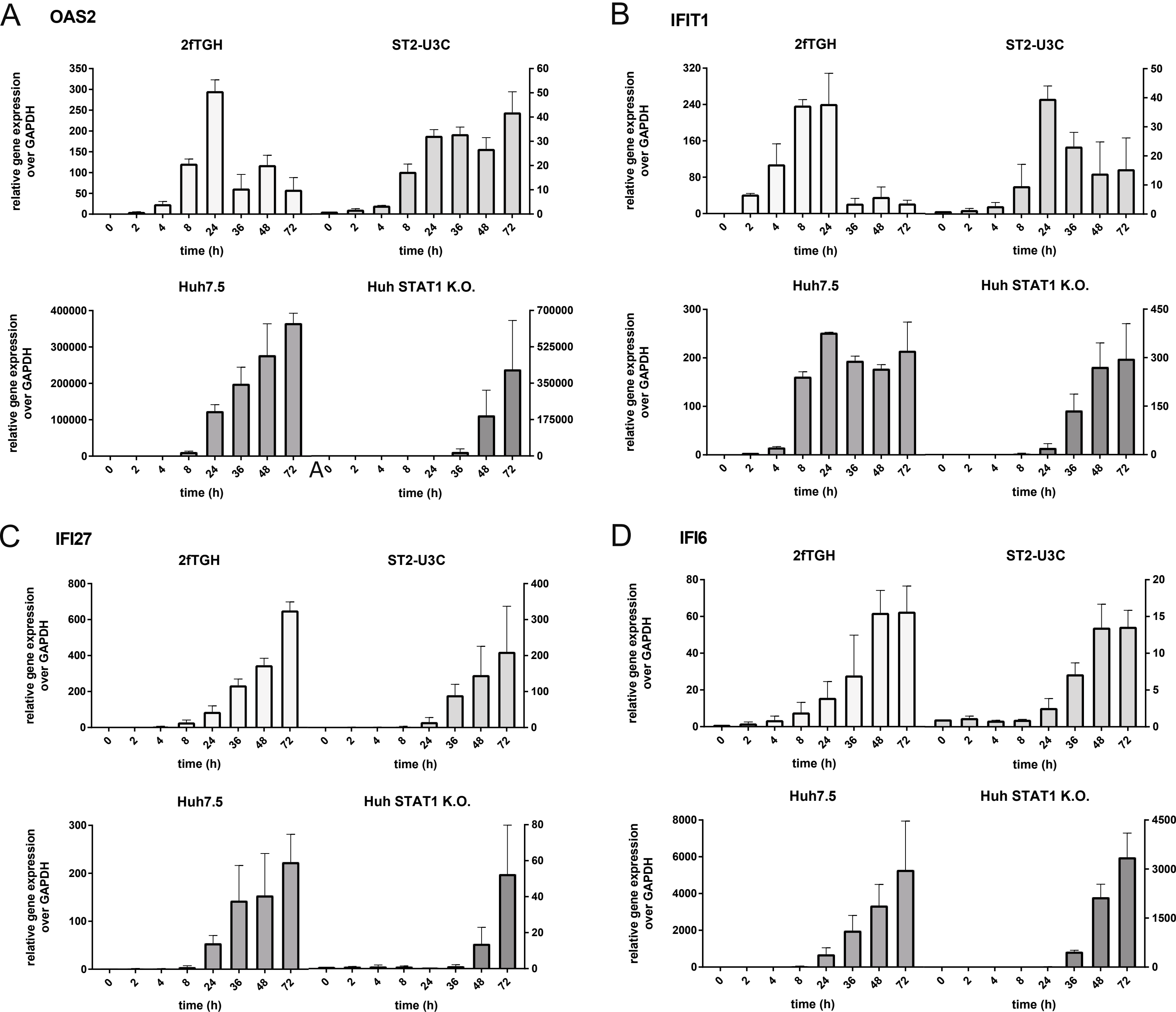

SUPPLEMENTARY FIGURE S2

ISRE HOMER motif 178

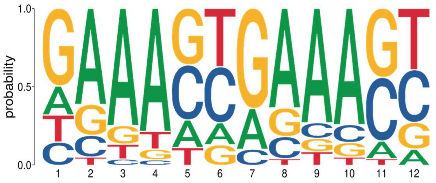

GAS HOMER motif 354

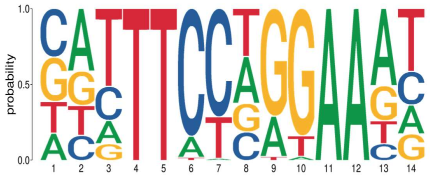

ISRE HOMER motif 179

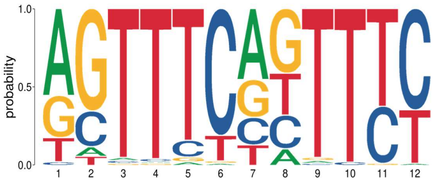

GAS HOMER motif 356

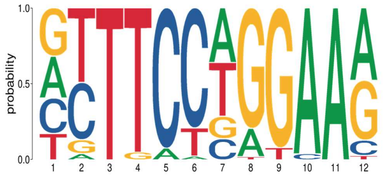

ISRE HOMER motif 181

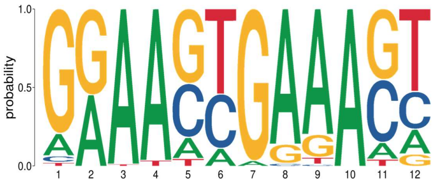

GAS HOMER motif 356

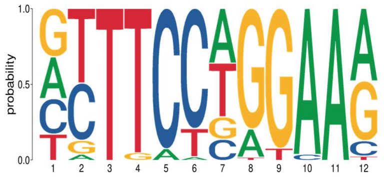

GAS HOMER deNovo motif 4

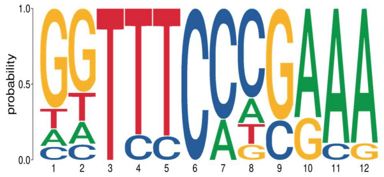

SUPPLEMENTARY FIGURE S3

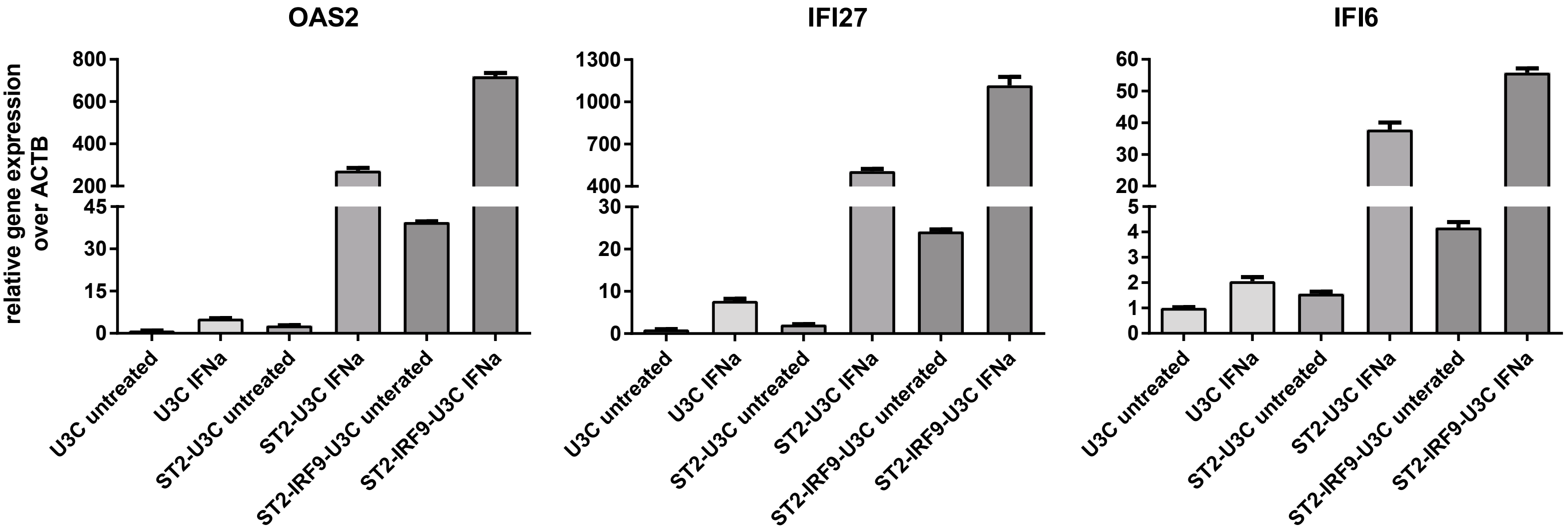

Table S1. Binding profiles statistical analysis.

2fTGH

group1	group2	p	p.adj	p.format	p.signif
UN_STAT1	2h_STAT1	3,77E-07	2,30E-06	3,80E-07	****
UN_STAT1	24h_STAT1	1,10E-04	3,30E-04	0,00011	***
UN_STAT1	72h_STAT1	1,02E-03	2,00E-03	0,00102	**
2h_STAT1	24h_STAT1	5,48E-06	2,20E-05	5,50E-06	****
2h_STAT1	72h_STAT1	1,52E-06	7,60E-06	1,50E-06	****
24h_STAT1	72h_STAT1	6,56E-02	6,60E-02	0,06563	ns
UN_STAT2	2h_STAT2	8,82E-06	5,30E-05	8,80E-06	****
UN_STAT2	24h_STAT2	5,66E-04	1,10E-03	0,00057	***
UN_STAT2	72h_STAT2	1,16E-05	5,80E-05	1,20E-05	****
2h_STAT2	24h_STAT2	1,71E-05	6,80E-05	1,70E-05	****
2h_STAT2	72h_STAT2	5,16E-05	1,50E-04	5,20E-05	****
24h_STAT2	72h_STAT2	7,82E-03	7,80E-03	0,00782	**
UN_IRF9	2h_IRF9	1,29E-04	5,20E-04	0,00013	***
UN_IRF9	24h_IRF9	6,00E-06	3,00E-05	6,00E-06	****
UN_IRF9	72h_IRF9	6,42E-08	3,90E-07	6,40E-08	****
2h_IRF9	24h_IRF9	1,04E-03	3,10E-03	0,00104	**
2h_IRF9	72h_IRF9	1,67E-03	3,30E-03	0,00167	**
24h_IRF9	72h_IRF9	3,63E-01	3,60E-01	0,36336	ns
UN_pSTAT1	2h_pSTAT1	4,16E-06	1,70E-05	4,20E-06	****
UN_pSTAT1	24h_pSTAT1	1,58E-06	7,90E-06	1,60E-06	****
UN_pSTAT1	72h_pSTAT1	1,21E-06	7,30E-06	1,20E-06	****
2h_pSTAT1	24h_pSTAT1	2,25E-02	6,70E-02	0,022	*
2h_pSTAT1	72h_pSTAT1	3,21E-02	6,70E-02	0,032	*
24h_pSTAT1	72h_pSTAT1	8,34E-01	8,30E-01	0,834	ns
UN_pSTAT2	2h_pSTAT2	1,83E-05	7,30E-05	1,80E-05	****
UN_pSTAT2	24h_pSTAT2	3,21E-06	1,60E-05	3,20E-06	****
UN_pSTAT2	72h_pSTAT2	2,10E-06	1,30E-05	2,10E-06	****
2h_pSTAT2	24h_pSTAT2	1,87E-02	5,60E-02	0,019	*
2h_pSTAT2	72h_pSTAT2	2,16E-01	2,50E-01	0,216	ns
24h_pSTAT2	72h_pSTAT2	1,25E-01	2,50E-01	0,125	ns

Huh7.5

group1	group2	p	p.adj	p.format	p.signif
UN_STAT1	2h_STAT1	1,55E-07	6,20E-07	1,50E-07	****
UN_STAT1	24h_STAT1	2,35E-08	1,20E-07	2,40E-08	****
UN_STAT1	72h_STAT1	8,51E-09	5,10E-08	8,50E-09	****
2h_STAT1	24h_STAT1	1,39E-03	2,80E-03	0,00139	**
2h_STAT1	72h_STAT1	1,07E-04	3,20E-04	0,00011	***
24h_STAT1	72h_STAT1	1,22E-01	1,20E-01	0,12201	ns
UN_STAT2	2h_STAT2	4,00E-10	2,40E-09	4,00E-10	****
UN_STAT2	24h_STAT2	1,07E-08	5,30E-08	1,10E-08	****
UN_STAT2	72h_STAT2	1,87E-08	7,50E-08	1,90E-08	****
2h_STAT2	24h_STAT2	3,63E-03	1,10E-02	0,0036	**
2h_STAT2	72h_STAT2	3,66E-02	7,30E-02	0,0366	*
24h_STAT2	72h_STAT2	3,81E-01	3,80E-01	0,3812	ns
UN_IRF9	2h_IRF9	7,00E-09	4,20E-08	7,00E-09	****
UN_IRF9	24h_IRF9	2,01E-08	1,00E-07	2,00E-08	****
UN_IRF9	72h_IRF9	2,17E-08	1,00E-07	2,20E-08	****
2h_IRF9	24h_IRF9	4,95E-01	4,90E-01	0,495	ns
2h_IRF9	72h_IRF9	5,46E-02	1,60E-01	0,055	ns
24h_IRF9	72h_IRF9	2,07E-01	4,10E-01	0,207	ns
UN_pSTAT1	2h_pSTAT1	1,00E-08	6,00E-08	1,00E-08	****
UN_pSTAT1	24h_pSTAT1	1,20E-08	6,00E-08	1,20E-08	****
UN_pSTAT1	72h_pSTAT1	1,51E-08	6,00E-08	1,50E-08	****
2h_pSTAT1	24h_pSTAT1	1,42E-04	4,30E-04	0,00014	***
2h_pSTAT1	72h_pSTAT1	6,61E-04	1,30E-03	0,00066	***
24h_pSTAT1	72h_pSTAT1	4,77E-01	4,80E-01	0,47719	ns
UN_pSTAT2	2h_pSTAT2	5,08E-10	3,00E-09	5,10E-10	****
UN_pSTAT2	24h_pSTAT2	1,83E-08	9,20E-08	1,80E-08	****
UN_pSTAT2	72h_pSTAT2	2,72E-08	1,10E-07	2,70E-08	****
2h_pSTAT2	24h_pSTAT2	3,77E-04	1,10E-03	0,00038	***
2h_pSTAT2	72h_pSTAT2	2,37E-02	4,70E-02	0,02369	*
24h_pSTAT2	72h_pSTAT2	6,96E-02	7,00E-02	0,0696	ns

ST2-U3C

group1	group2	p	p.adj	p.format	p.signif
UN_STAT2	2h_STAT2	1,45E-08	7,30E-08	1,50E-08	****
UN_STAT2	24h_STAT2	1,27E-01	2,50E-01	0,13	ns
UN_STAT2	72h_STAT2	4,20E-09	2,50E-08	4,20E-09	****
2h_STAT2	24h_STAT2	1,52E-07	4,60E-07	1,50E-07	****
2h_STAT2	72h_STAT2	3,61E-01	3,60E-01	0,36	ns
24h_STAT2	72h_STAT2	1,02E-07	4,10E-07	1,00E-07	****
UN_IRF9	2h_IRF9	6,80E-05	2,70E-04	6,80E-05	****
UN_IRF9	24h_IRF9	2,95E-05	1,50E-04	3,00E-05	****
UN_IRF9	72h_IRF9	6,70E-10	4,00E-09	6,70E-10	****
2h_IRF9	24h_IRF9	8,74E-01	8,70E-01	0,8738	ns
2h_IRF9	72h_IRF9	3,63E-03	1,10E-02	0,0036	**
24h_IRF9	72h_IRF9	5,47E-03	1,10E-02	0,0055	**
UN_pSTAT2	2h_pSTAT2	1,80E-09	1,10E-08	1,80E-09	****
UN_pSTAT2	24h_pSTAT2	7,30E-09	2,90E-08	7,30E-09	****
UN_pSTAT2	72h_pSTAT2	1,78E-09	1,10E-08	1,80E-09	****
2h_pSTAT2	24h_pSTAT2	6,54E-01	1,00E+00	0,65	ns
2h_pSTAT2	72h_pSTAT2	3,21E-01	9,60E-01	0,32	ns
24h_pSTAT2	72h_pSTAT2	5,99E-01	1,00E+00	0,6	ns

Huh STAT1KO

group1	group2	p	p.adj	p.format	p.signif
UN_STAT2	2h_STAT2	1,84E-03	3,70E-03	0,0018	**
UN_STAT2	24h_STAT2	3,21E-08	1,30E-07	3,20E-08	****
UN_STAT2	72h_STAT2	3,96E-11	2,40E-10	4,00E-11	****
2h_STAT2	24h_STAT2	6,27E-07	1,90E-06	6,30E-07	****
2h_STAT2	72h_STAT2	4,00E-09	2,00E-08	4,00E-09	****
24h_STAT2	72h_STAT2	4,95E-01	5,00E-01	0,495	ns
UN_IRF9	2h_IRF9	1,12E-03	2,20E-03	0,0011	**
UN_IRF9	24h_IRF9	1,36E-08	5,40E-08	1,40E-08	****
UN_IRF9	72h_IRF9	1,80E-11	1,10E-10	1,80E-11	****
2h_IRF9	24h_IRF9	5,17E-08	1,60E-07	5,20E-08	****
2h_IRF9	72h_IRF9	7,92E-11	4,00E-10	7,90E-11	****
24h_IRF9	72h_IRF9	5,10E-01	5,10E-01	0,5104	ns
UN_pSTAT2	2h_pSTAT2	1,88E-03	3,80E-03	0,0019	**
UN_pSTAT2	24h_pSTAT2	2,98E-08	1,20E-07	3,00E-08	****
UN_pSTAT2	72h_pSTAT2	2,88E-11	1,70E-10	2,90E-11	****
2h_pSTAT2	24h_pSTAT2	7,71E-07	2,30E-06	7,70E-07	****
2h_pSTAT2	72h_pSTAT2	1,26E-08	6,30E-08	1,30E-08	****
24h_pSTAT2	72h_pSTAT2	1,83E-01	1,80E-01	0,1831	ns

Table S2. Genes commonly upregulated in 2fTGH vs Huh7.5 (WTs, $log_2FC>=1$) and ST1-ST2-IRF9-U3C ($log_2FC>=0,5$).

Gene name	Log_2FC	padj
APOL1	1,179548695	0,004684412
APOL6	1,004077196	0,038984999
BST2	6,135203905	1,35927E-50
C1R	1,408098465	0,031410471
CASP1	1,910053199	0,015147452
DDX60	1,821430803	0,000316359
DENND2D	2,789095461	0,048039125
DTX3L	1,352686939	0,000116872
EIF2AK2	1,52332203	3,34411E-07
ERAP1	0,965079469	0,000499671
ERAP2	1,130778979	0,003692999
HELZ2	0,96070728	0,014920517
HERC6	1,618186178	0,000600321
HLA-B	1,178511842	0,001660318
HLA-C	1,456434161	1,75465E-07
IFI27	4,788538368	0,000413159
IFI6	4,034905117	3,7793E-25
IFIT1	3,212109227	1,06397E-13
IFIT2	2,829436266	5,97437E-05
IFIT3	1,906823009	4,21176E-05
IFITM1	2,733004839	5,38304E-20
IFITM3	0,752739796	0,007072815
IRF9	7,842346026	1,87668E-80
ISG15	1,562830472	2,90124E-05
NRP2	0,870136135	0,002302707
OAS1	1,471623318	0,002759109
OAS2	4,639318222	1,20916E-26
OAS3	0,941039617	0,028400103
PARP10	1,009511582	0,001614949
PARP14	1,66043833	3,31686E-08
PARP9	1,619836156	4,81902E-05
PDGFRL	2,314318252	0,013940482
RTP4	3,339158993	0,001009013
SAMHD1	1,284115233	0,009206662
STAT1	3,374870457	2,50965E-06
STAT2	5,052303916	1,9208E-92
THEMIS2	1,173305564	0,035610056
TRANK1	2,347593899	1,32527E-06
UBA7	1,029286496	0,013001475
UBE2L6	1,165570135	0,00053531
USP18	3,111223801	4,5583E-06
XAF1	3,694263817	1,24333E-05
ZBTB42	1,070772287	0,031039847

Table S3. qPCR primers.

Gene name	Primer sequence	
	Forward	Reverse
GAPDH	CAATATGATTCCACCCATGGCAA	GATCTCGCTCCTGGAAGATGG
IFI27	GTCACTGGGAGCAACTGGAC	GGGCAGGGAGCTAGTAGAAC
IFI6	ATCCTGAATGGGGGCGG	AGATACTTGTGGGTGGCGTAG
OAS2	CAATCAGCGAGGCCAGTAAT	TCCAGGTTGGGAGAAGTCAA
IFIT1	CTTGCAGGAAACACCCACTT	CCTCTAGGCTGCCCTTTTGT

Table S4. ChIP-PCR primers.

Gene name	Primer sequence	
	Forward	Reverse
NANOG	TGGTAGACGGGATTAACTGAG	GAAGGCTCTATCACCTTAGA
OAS2	CGCTGCAGTGGGTGGAGAGA	GCCGGCAAGACAGTGAATGG
IFI27	CTTCTGGACTGCGCATGAGG	CCACCCCGACTGAAGCACTG
IFIT1	GCAGGAATTCCGCTAGCTTT	GCTAAACAGCAGCCAATGGT
ISG15	AGGGAAACCGAAACTGAAGC	TGAGGCACACACGTCAGG
STAT1	CGCTCAGCCAATTAGACGC	GTAAACAGAACGCCAGTTCCC
STAT2	TGTCACCAAGCAGGCTGTC	TCTGTTCTGTTAGGCTCAGGC
IRF9	AGATGCTGCTGCCCTCTAGT	CCCCTTTCTACAGTCCCCA
